# Low performance of Policimbac® broth microdilution in determining polymyxin B MIC for *Klebsiella pneumoniae*

**DOI:** 10.3389/fcimb.2023.1139784

**Published:** 2023-05-30

**Authors:** Natália Conceição Rocha, Jessica Mariana Lopes, Karolayne Larissa Russi, Jussara Kasuko Palmeiro, Raquel Girardello

**Affiliations:** ^1^ Laboratório de Microbiologia Molecular e Clínica, Programa de Pós-Graduação em Ciências da Saúde, Universidade São Francisco, Bragança Paulista, Brazil; ^2^ Laboratório de Microbiologia, Hospital Universitário São Francisco na Providência de Deus, Bragança Paulista, Brazil; ^3^ Laboratório de Microbiologia Molecular Aplicada (MiMA), Departamento de Análises Clínicas (ACL), Centro de Ciências da Saúde (CCS), Universidade Federal de Santa Catarina (UFSC), Florianópolis, Brazil

**Keywords:** polymyxin B, colistin, broth microdilution, minimal inhibitory concentration, *Klebsiella pneumoniae*

## Abstract

*Klebsiella pneumoniae* is a global threat to healthcare, and despite the availability of new drugs, polymyxins are still an important therapeutic option for this and other resistant gram-negative pathogens. Broth microdilution is the only method that is recommended for polymyxins. In this study, we evaluated the accuracy of a commercial Policimbac® plate in determining the polymyxin B MIC for *K. pneumoniae* clinical isolates. The results were compared with those of the broth microdilution method according to ISO 16782. The Policimbac® plate had an excellent 98.04% categorical agreement, but unacceptable 31.37% essential agreement rates. Almost 2% of major errors as observed. Additionally, 52.94% of the strains overestimated the MIC at 1 µg/mL. Three isolates were excluded from the analysis due to the drying of the Policimbac® plate. To avoid dryness, we included wet gauze for the test, obtaining a 100% of categorical agreement rate; however, a low essential agreement was maintained (25.49%). In conclusion, the Policimbac® plate was unable to correctly determine the polymyxin B MIC for *K. pneumoniae* isolates. This low performance may interfere with the clinical use of the drug and, thus, with the result of the patient’s treatment.

## 1 Introduction

*Klebsiella pneumoniae* is a global threat to healthcare, with mortality rates ranging from 40 to 70% in patients with bloodstream infections. Despite the availability of new drugs in the last few years, mainly for KPC-producing *K. pneumoniae* in Brazil and other countries, polymyxins are still an important therapeutic option for this and other resistant gram-negative pathogens. New cephalosporins associated with beta-lactamase inhibitors developed for the treatment of KPC-producing *K. pneumoniae* are not available for all hospitals in Brazil due to their high cost. Additionally, resistance to ceftazidime-avibactam has been reported (Ezadi et al., 2019; Jiang et al., 2022) and an increase in NDM-producing *K. pneumoniae* dissemination has been reported after COVID-19 (Ministério da Saúde, 2022).

Susceptibility tests for polymyxins have been a challenge for routine clinical laboratories because of the low accuracy and reproducibility of the available methods (Ezadi et al., 2019; Jiang et al., 2022). The regulatory committees for antimicrobial susceptibility tests recommend only the broth microdilution method for polymyxin tests. Because broth microdilution is a laborious and expensive method to perform in the clinical routine, several laboratories choose a commercial plate. According to the BR-GLASS Global Antimicrobial Resistance Surveillance System (Pillonetto et al., 2020), it was not possible to analyze the rates of resistance to polymyxins in Brazil because a few laboratories use the recommended method for this test. Policimbac® (Probac do Brasil) is a commercially available polymyxin B lyophilized microdilution plate that is more frequently used by laboratories in Brazil.

In this study, we evaluated the accuracy of a commercial Policimbac® plate and proposed an adaptation to improve the performance for clinical isolates of *K. pneumoniae.*

## 2 Materials and methods

### 2.1 Cation-adjusted broth microdilution

Cation-adjusted broth microdilution was used as gold standard method. The plates were prepared according to ISO16782. The clinical isolates included in this study were previously identified as *K. pneumoniae* by Vitek-MS (Biomerriéux). The strains were isolated from urinary tract infections (13 isolates), surveillance samples (9 isolates), soft tissue infections (9 isolates), surgical infections (8 isolates), bloodstream infections (8 isolates), and pneumonia (7 isolates). All strains exhibited multidrug-resistance profile and carbapenem-resistant phenotypes due to KPC production. The polymyxin B susceptible isolates were stored at Microbiology Laboratory from Universidade São Francisco. The polymyxin B resistant isolates were kindly provided by Dr Ana Cristina Gales, from Universidade Federal de São Paulo, Brazil, and Dr Nilton Lincopan, from Universidade de São Paulo. Briefly, a cation adjusted broth microdilution plate was prepared twice concentrate to obtain a final concentration of 0.006 to 64 µg/mL of polymyxin B sulfate salt (Sigma-Aldrich). Bacterial inoculums were prepared at 0.5 McFarland scale (1.5 x 10^8^ CFU) in 0,75% saline solution sterile and diluted to 5 x 10^6^ CFU in cation adjusted broth Mueller-Hinton Broth (Sigma-Aldrich). One hundred microliters were added to broth microdilution plate. *Escherichia coli* ATCC 25922 and *Pseudomonas aeruginosa* ATCC 27853 were used as quality control. The plates were incubated at 37°C (+/- 2°C) for 18 to 24 hours and the results were read by visual inspection. The MIC was considered the first well where do not observe bacterial growth.

### 2.2 Policimbac® broth microdilution

The Policimbac® plate was initially used according to the manufacturer’s recommendations (Jaguaribe et al., 2016). A 0,5 McFarland scale inoculum (1.5 x 10^8^ CFU) was prepared in 0.75% NaCl solution (Tube 1) followed by two dilutions: 1.5 x 10^6^ in a Tube 2, and 1.5 x 10^8^ a Tube 3. The NaCl solution was not provided by the manufacturer. One hundred microliters from Tube 3 were added to lyophilized broth microdilution plate, which was returned to the original packing before being incubated for 18h to 24h at 37°C (+/- 2°C). After the incubation period, a drop of disclosure solution (provided in the kit) was added in the well`s plate. The plates were returned to 37°C (+/- 2°C) by 20 minutes before results interpretation. Red colors were observed in the wells which there was bacterial growth. The results were interpreted according to EUCAST (2022) breakpoints.

In the second step, sterile gauze moistened with sterile water was added to the package to avoid drying the plate.

### 2.3 Data analysis

The results were interpreted according to EUCAST breakpoints (The European Committee on Antimicrobial Susceptibility Testing, 2022). Categorical Agreement (CA), Essential Agreement (EA), Major Errors (ME), and Very Major Errors (VME) were evaluated according to FDA recommendations (U.S. Food and Drug Administration, 2021). The CA represents the same susceptibility or resistance category when comparing the Policimbac and gold standard broth microdilution, while EA represent the MIC agreement (+/- 1log_2_), between both methods. ME and VME represent false resistance and false susceptibility, respectively.

## 3 Results

**Figure 1:**
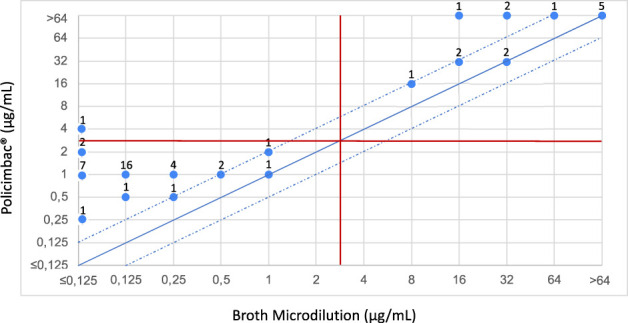
Scattergram showing the agreement between the broth microdilution tests and commercial Policimbac® plate, using protocol performed according to manufacturer’s recommendations. The solid diagonal blue line corresponds to the agreement of MICs between the two methodologies. The two dotted diagonal blue lines correspond to one log above and one log below the MIC of agreement, which is still considered to be within the acceptable limit of EA. The horizontal and vertical red lines correspond to the susceptibility category breakpoints for polymyxins, according to EUCAST. The numbers distributed in the points correspond to the quantity of strains that were classified in these MICs, in the methodologies tested.

Fifty-four clinical isolates of *K. pneumoniae* were tested, according to the manufacturer’s recommendations. Three of the isolates were excluded from the analysis due to drying of the Policimbac® plate, and 51 isolates were analyzed. The Policimbac® plate had an excellent 98.04% CA but an unacceptable 31.37% EA rates. Low EA occurred because the strains were between 1 and 6 logs higher than those obtained by the reference broth microdilution method. In this way, 1.96% ME was observed, and no VME was reported (**Figure 1**). In our tests, 27 of the 51 isolates (52.94%) were incorrectly classified at an MIC of 1 µg/mL. These isolates had MICs varying from ≤ 0.125 to 0.5 µg/mL according to the reference method (**Figure 1**).

**Figure 2:**
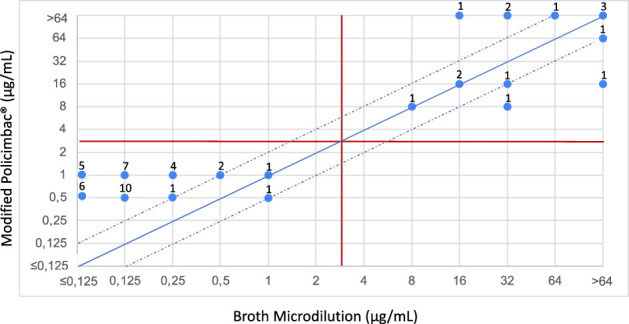
Scattergram showing the agreement between the broth microdilution tests and commercial Policimbac^®^ plate, using modified protocol performed adding a moistened gauze during the incubation period. The solid diagonal blue line corresponds to the agreement of MICs between the two methodologies. The two dotted diagonal blue lines correspond to one log above and one log below the MIC of agreement, which is still considered to be within the acceptable limit of EA. The horizontal and vertical red lines correspond to the susceptibility category breakpoints for polymyxins, according to EUCAST. The numbers distributed in the points correspond to the quantity of strains that were classified in these MICs, in the methodologies tested.

In a second experiment, to avoid wasting the plates due to dryness, the same 54 strains were incubated again, including a wet gauze, in the package. Three of the strains had to be excluded from the analysis due to the “Skipped Well” effect, that is common to polymyxins microdilution (EUCAST, 2019) and 51 isolates were also analyzed. The addition of wet gauze prevents drying of the plate and slightly improves the performance of the method. The modified protocol achieved a 100% CA rate; however, a low EA was maintained (25.49%). No ME or VME was observed using the modified protocol (**Figure 2**). Additionally, the results were read in the presence and absence of colorimetric reagents in the plate, but no differences were observed.

In all tests, the quality controls *E. coli* ATCC 25922 and *P. aeruginosa* ATCC 27853 had MIC in the superior limit (1 µg/mL and 2 µg/mL, respectively) in the Policimbac®. Commonly, the *E. coli* ATCC 25922 has MIC in the lower limit (0.5 µg/mL) using the gold standard method. Despite that, both quality controls remain in the range recommended for EUCAST (0.5 – 1 µg/mL for *E. coli* ATCC 25922 and 1 – 2 µg/mL for *P. aeruginosa* ATCC 27853). On the other hand, the *mcr-1* positive NCTC 13846 (4 µg/mL), varied between 8 and 16 µg/mL in the Policimbac®, remaining above the range recommended by EUCAST (2 – 8 µg/mL).

## 4 Discussion

The polymyxins susceptibility test is a challenge for clinical laboratories worldwide. Disk diffusion, Etest and automated methods are not recommended due to high error rates observed (Ezadi et al., 2019; Pogue et al., 2019). Manual broth microdilution is the method currently recommended and manufacturer’s plates are commercially available. Policimbac® is one of the main manufactured polymyxin B plate used in Brazil and their inaccuracy represent a significant problem, as polymyxin B is still an important therapeutic choice for multidrug-resistant Gram-negative isolates, including *K. pneumoniae*. KPC-producing *K. pneumoniae* is the first Gram-negative pathogen associated to bloodstream infection in patients in intensive therapy units from Brazil (Gomes et al., 2021). *K. pneumoniae* clinical isolates commonly do not present borderline MICs for polymyxins, in this way, a high categorical agreement and absence of very major errors was observed in our study. A previous study from Dalmolin et al., 2020 evaluate the same commercial plate and found similar results, however, despite low essential agreement observed, the authors do not alert to clinical problem involving this phenomenon and suggest that the method is useful. It’s important to highlight that, despite the absence of Very Major Errors in our study, the low essential agreement rate and high percentage of strains being incorrectly classified in MIC 1 µg/mL may represent a clinical problem for patient treatment (Tsuji et al., 2019; Turnidge et al., 2019). The International Consensus Guidelines for the Optimal Use of Polymyxins affirm that due to inaccuracies in polymyxin susceptibility testing, relying on MIC may lead to suboptimal exposures, resulting in resistance and therapeutic failure (Tsuji et al., 2019). The consensus recommends that patients infected by isolates with MIC varying between 0.125 - 0.5 µg/mL can receive 2 mg/kg/day of drug, while isolates with MIC between 1 - 2 µg/mL should receive dosages between 2.5 - 3 mg/kg/day. High-concentration usage based on an incorrectly overestimated MIC may increase the incidence and severity of acute kidney injury (Tsuji et al., 2019). The elevated rate of overestimated in MIC 1 µg/mL represent increased drug dosage used for patients that should receive reduced dosage. The Policimbac® plate is not provided with a lid, and the protocol recommends incubation of the plate in the original package. Due to the absence of a lid, the plate is incubated open, and consequently, dryness occurs. This dryness may also be the cause of low EA observed. The use of wet gauze improves the performance of the test, as no ME and VME were observed; however, it was not sufficient to solve the problem of low EA. Finally, the Policimbac® protocol requires three dilutions in saline solution, which are not provided by the manufacturer. This may be a pitfall of testing in a large laboratory. In addition, the quality controls strains recommended by antimicrobial susceptibility tests committees generally have MICs elevated but remain in the range recommended. This makes it difficult to observe errors of the method in the clinical routine. It’s important to highlight that the low number of strains evaluated, and the lack of borderline MICs were the limitations of this study, since only 17 polymyxin B resistant isolates was initially available to test, and no borderline MIC was observed. For *K. pneumoniae* clinical isolates are not commonly found borderline MICs. Despite recent drugs commercially available, mainly for KPC-producing *K. pneumoniae*, polymyxins is still one of the main therapeutic options for this specie and other Gram-negative bacilli. The Covid-19 pandemic change the scenery of resistance worldwide and increase of NDM-producing *K. pneumoniae* strains were observed (Arend et al., 2023). So, the polymyxins remain as one of the last therapeutic options available and the laboratories should be prepared to offer a correct MIC.

## 5 Conclusion

In conclusion, despite the high CA shown by Policimbac®, their low performance in determining polymyxin B MIC results in a clinical problem for therapeutic use. Polymyxins are still used in Brazil and some countries, manly after the Covid-19, and the correct MIC determination is needed to improve their clinical use and reduce the mortality rates due to carbapenem-resistant *K. pneumoniae* isolates.

## Data availability statement

The original contributions presented in the study are included in the article/supplementary material. Further inquiries can be directed to the corresponding author.

## Author contributions

NR contributed to the study design, conduct of experiments, data analysis, and writing of the manuscript. JL and KR contributed to the conduction of experiments. JP contributed to data analysis and revised the manuscript. RG contributed to study design, conduction of experiments, data analysis, and writing and revision of the manuscript. All authors contributed to the article and approved the submitted version.
